# Risk Factors and Prognosis of Early Neurological Deterioration after Bridging Therapy

**DOI:** 10.2174/0115672026287986240104074006

**Published:** 2024-01-17

**Authors:** Yiju Xie, Shengyu Li, Liuyu Liu, Shiting Tang, Yayuan Liu, Shuangquan Tan, Zhijian Liang

**Affiliations:** 1 Department of Neurology, The First Affiliated Hospital of Guangxi Medical University, Nanning, Guangxi Province, China;; 2 Department of Neurology, Wuming Hospital of Guangxi Medical University, Nanning, Guangxi Province, China;; 3 Department of Neurology, The second Affiliated Hospital of Guangxi Medical University, Nanning, Guangxi Province, China;; 4 Department of Neurology, The First Affiliated Hospital, Sun Yat-sen University, Guangzhou, Guangdong Province, China

**Keywords:** Early neurological deterioration, acute ischemic stroke, bridging therapy, risk factors, prognosis, systolic blood pressure

## Abstract

**Background:**

Early neurological deterioration (END) after bridging therapy (BT) of acute ischemic stroke (AIS) patients is associated with poor outcomes.

**Objective:**

We aimed to study the incidence, risk factors and prognosis of END after BT.

**Methods:**

From January to December 2021, the clinical data of AIS patients treated by BT (intravenous thrombolysis with alteplase prior to mechanical thrombectomy) from three comprehensive stroke centers were analyzed. Patients were divided into non-END group and END group according to whether they developed END within 72 hours of symptom onset. Modified Rankin scale (mRS) was used to assess the patient’s prognosis at 90 days, and favorable outcomes were defined as mRS≤2. The incidence of END was investigated, and binary logistic regression analysis was used to explore its associated factors.

**Results:**

The incidence of END after BT was 33.67%. The eligible 90 patients included 29 cases in the END group and 61 cases in the non-END group. Multivariate Logistic regression analysis showed that increase of systolic blood pressure (SBP) (OR=1.026, 95%CI:1.001-1.051, *p* =0.043), higher level of blood glucose at admission (OR=1.389, 95%CI:1.092-1.176, *p* =0.007) and large artery atherosclerosis (LAA) subtype (OR=8.009, 95%CI:2.357-27.223, *p* =0.001) were independent risk factors of END. Compared with the non-END group, the END group had significantly lower rates of good outcomes (6.90% *versus* 65.57%, *p* =0.001) while higher rates of mortality (44.83% *versus* 4.92%, *p* =0.001).

**Conclusion:**

It was found that the incidence of END after BT in AIS patients was 33.67%. An increase in SBP, higher glucose levels at admission, and LAA were independent risk factors of END that predicted a poor prognosis.

## 1 INTRODUCTION

The phenomenon that some acute ischemic stroke (AIS) patients do not recover or even deteriorate in the short term after treatment is so-called early neurological deterioration (END) [1]. Observed rates of END have been variable, and up to 32.8% of patients experienced END after intravenous thrombolysis (IVT) [2], while the incidence of END after mechanical thrombectomy (MT) is as high as 51.4% [3]. END was significantly associated with poor prognosis [4], and research on the risk factors of END will be helpful to the prevention and treatment of it. Current studies have found that hyperglycemia, admission of high systolic blood pressure (SBP), presence of diabetes mellitus, prior transient ischemic attacks (TIA) or stroke, the stroke subtype of large artery atherosclerosis (LAA) increased the risk of END after IVT [5-9]. The risk factors of END after MT varied according to the causes of deterioration. Inclusions within 24 hours after MT were associated with age, internal carotid artery occlusion and the number of passes [4], while patients with higher levels of blood glucose, thrombin time (TT) at admission and National Institutes of Health Stroke Scale (NIHSS) score after the operation are more likely to develop symptomatic intracranial hemorrhage (sICH) [10]. Bridging therapy (BT), IVT with alteplase prior to MT is the first recommended treatment for stroke patients with acute large vessel occlusion (LVO) within 4.5 hours of symptom onset [11]. Data showed that 27.00%-59.52% of LVO stroke patients received BT [12, 13], and END still occurs in some of them. Studies that specifically focused on the risk factors of END after BT are rare. Hence, the aim of our study is to investigate the incidence, risk factors, and prognosis of END after BT preliminarily.

## 2 MATERIALS AND METHODS

### 2.1 Patient Inclusion

We conducted a retrospective analysis of the clinical data of AIS patients treated by BT (IVT with alteplase prior to MT) at three comprehensive stroke centers (First Affiliated Hospital of Guangxi Medical University, Wuming Hospital of Guangxi Medical University, The Second Affiliated Hospital of Guangxi Medical University) from January to December 2021. Written informed consent was waived, given the retrospective nature of the study. All patients received BT, and medical management was based on the current guidelines for acute stroke management [14]. Eligible patients were divided into non-END group and END group according to whether they developed END within 72 hours of symptom onset, and the early neurological status of each patient after operation was reviewed in detail. Inclusion criteria were as follows: age≥ 18 years; the diagnosis of AIS and the treatment of IVT with alteplase met the criteria of the guidelines; the treatment of MT met the standard procedures of endovascular treatment of the guidelines. Exclusion criteria were as follows: the dose of IVT with alteplase did not meet the standard dose; END occurred during IVT; modified Rankin scale (mRS)>2 at 90 days due to recurrent stroke or other causes; history of severe organ failure or malignant tumor; failure to following or insufficient information.

### 2.2 Data Collection and Assessment

Baseline demographic and clinical information for all enrolled patients included age, gender, and medical history (hypertension, diabetes mellitus, coronary artery disease, atrial fibrillation, prior TIA or stroke, current smoking). Laboratory findings included blood glucose, white blood cells, platelets, fibrinogen, international normalized ratio (INR), prothrombin time (PT), TT, total cholesterol (TC), triglycerides (TG), high-density lipoprotein (HDL), low-density lipoprotein (LDL), homocysteine (HCY) at admission. Clinical findings included SBP, diastolic blood pressure (DBP), NIHSS score at admission, TOAST classification, site of vascular territories, symptom onset to thrombolysis time (OTT), symptom onset to groin puncture time (OTP), symptom onset to first recanalization time (OTR), puncture to first recanalization time (PTR); mTICI at the end of the procedure.

END was defined as an increase of ≥4 points in the baseline NIHSS score within 72 hours of symptom onset [1]. Stroke subtype was determined by Trial of Org 10172 in the Acute Stroke Treatment classification [15]. Ischemic stroke was classified as anterior circulation stroke (ACS) and posterior circulation stroke (PCS) according to the vascular territories in which infarction occurs [16]. Successful recanalization was defined as an mTICI score of 2b or 3 [17]. Clinical outcomes at 90 days included favorable (mRS≤2) and poor (mRS>2) outcomes. Thereinto, death was defined as mRS =6 [18].

### 2.3 Statistical analysis

Continuous variables were presented as mean with standard deviation (SD) or median with interquartile range and analyzed with Student t-test or Mann-Whitney U test according to the normality of data distribution. Categorical variables were described as counts (percentages) and were compared using Pearson χ^2^ or Fisher exact tests. Binary logistic regression analysis was performed to assess the risk factors of END after BT. To adjust for potential confounders, variables with *p* <0.05 in univariate analysis were entered into multivariable analysis with forward stepwise method and the odds ratios (OR) and 95% confidence intervals (CI) were presented. Two-tailed *p* <0.05 was considered statistically significant. The statistical software is SPSS 20.0 (SPSS, IBM).

## 3 RESULTS

### 3.1 Patients’ Demographic and Clinical Characteristics

A total of 98 AIS patients underwent BT, and 33 cases (33.67%) suffered from END.8 cases were excluded based on the inclusion and exclusion criteria. The remaining 90 patients were enrolled in the present study, including 29 cases in the END group and 61 cases in the non-END group. The study population flowchart is shown in Fig. (**1**).

Among the 90 eligible patients, the mean age was 63±13 years, aged from 36 to 89 years, and 54 (59.34%) cases were males. The most frequent stroke subtype was LAA, with 67 cases (73.63%), while the most prevalent affected vascular territory was ACS, with 72 cases (79.12%). There were 22 cases (30.56%) in ACS and 7 cases (38.89%) in PCS-developed END, respectively. Successful recanalization was achieved in 82 (91.11%) cases, and the median admission NIHSS score was 14. Compared with the non-END group, the age of the END group was older (67 years *versus* 62 years, *p* =0.043) with higher admission SBP (162 mmHg *versus* 150 mmHg, *p* =0.021) and blood glucose (7.0 mmol/L *versus* 6.1 mmol/L, *p* =0.012). AF was more prevalent in the END group than in the non-END group (41.38% *versus* 19.67%, *p* =0.030), whereas LAA was less common (58.62% *versus* 81.97%, *p* =0.018). There was no significant difference in other indicators between the two groups (Table **1**).

### 3.2 Risk Factors of END after Bridging Therapy

After adjustment for the confounding factors (age, LAA, history of atrial fibrillation, SBP, blood glucose at admission), increase of SBP (OR=1.026, 95%CI:1.001-1.051, *p* =0.043), higher level of blood glucose at admission (OR=1.389, 95%CI:1.092-1.176, *p* =0.007) and LAA subtype (OR=8.009, 95%CI:2.357-27.223, *p* =0.001) were independent risk factors of END after BT (Table **2**).

### 3.3 Clinical Outcomes at 90 Days of Non-END Group and END Group

Compared with the non-END group, the END group had significantly lower rates of good outcomes (6.90% *versus* 65.57%, *p* =0.001) while higher rates of mortality (44.83% *versus* 4.92%, *p* =0.001) (Table **3**).

## 4 DISCUSSION

END is not rare in AIS patients after reperfusion therapy and the rate of END after MT reported by studies varied. The rate of END in ACS after MT, with 18.8% in the previous report [19], was lower than that of 30.56% in the current study, which may be due to the lack of a standardized definition of END. We defined the time of END as within 72 hours of symptom onset, whereas they defined it as within 24 hours after endovascular thrombectomy in the previous study. Zhang *et al*. found that 40.2% of LVO stroke patients who received MT suffered from END [20], which was higher than that of 33.67% in our study. The main reason for the above difference is that the study population of the appeal studies were all the target patients treated by MT, including those who did not receive IVT. However, the present study was specifically focused on the patients who underwent MT after IVT with alteplase alone.

Blood pressure control is a key factor affecting the prognosis of AIS patients, and its management during the perioperative period of reperfusion therapy remains a clinical challenge [21]. When blood pressure fluctuates within a certain range, the body maintains a relatively constant cerebral blood flow by adjusting the diameter of cerebral small vessels. Cerebral autoregulation is the main way to prevent hypoperfusion or hyperperfusion of brain tissue [22]. A recent multicenter, retrospective case-control study showed a similar conclusion that admission SBP independently predicted END after MT [23]. Vilionskis *et al*. found that patients with SBP>180mmHg at admission had a significantly higher risk of neurological deterioration (ND) within 7 days after MT [24]. Powers *et al*. recommended that blood pressure should be actively controlled at SBP<180 mmHg, DBP < 110 mmHg before the reperfusion therapy [14] but they did not clarify a more precise range. In the present study, the mean admission SBP of the two groups were both high, 162mmHg in the END group and 150mmHg in the non-END group, respectively. Whether SBP control below 160mmHg is more reasonable for patients before BT needs further investigation.

Blood glucose is one of the risk factors of END that many studies have focused on. A study in 1995 showed that 26% of first-ever AIS patients experienced ND during treatment, and blood glucose at admission could independently predict ND [25]. Recently, Yang *et al*. applied an interpretable machine learning model to individually predict END in AIS patients treated with MT and observed that blood glucose was the most important variable [26]. Our results are concordant with those of some previous studies, but the mechanisms that how hyperglycemia induces ND after reperfusion therapy remain unclear. Some scholars proposed that hyperglycemia may aggravate ND, exacerbating blood-brain barrier dysfunction after ischemia-reperfusion injury by increasing oxidative stress and matrix metalloproteinase-9 (MMP-9) activity [27]. Desilles *et al*. put forward that hyperglycemia primes the thromboinflammatory cascade, amplifying downstream microthrombosis, which causes poor reperfusion and impaired neurological dysfunction [28]. However, Bevers *et al*. raised an alternative view that hyperglycemia is the result of nerve cell damage, not the cause. The loss of blood flow after ischemic stroke generates the disorder of cellular energy metabolism, following the inactivation of the sodium-potassium pump, which initiates the intracellular pathway and ultimately results in the toxic cell death of nerve cells and a rise in blood glucose [29].

The major cause of END after IVT is ischemia progression or recurrence from initial relevant arterial territory rather than complications such as intracranial hemorrhage or sICH, and LAA independently predicted the END caused by ischemic progression [7]. Coincidentally, Kim *et al*. classified the END mechanism after MT as ischemia progression, symptomatic hemorrhage and brain edema, and results revealed that LAA was a risk factor of END due to ischemic progression [18]. For stroke patients who received IVT prior to MT, we found that LAA still increased the risk of END at the present study. Inflammation mechanisms play an important role in the acute phase of stroke, and Nam *et al*. speculated that instability of vulnerable atherosclerotic plaques of the LAA subtype was more prone to frequent recurrence or progression, which results in a higher risk of END [30]. The safety and efficacy of direct mechanical thrombectomy (d-MT) *versus* BT in LVO stroke patients eligible for IVT has always been a topic of great interest in the clinical. Li *et al*. carried out a meta-analysis of observational studies and randomized controlled trials (RCTs) published up to October 30, 2021, and found that BT did not increase the risk of complications compared to d-MT [31]. However, another meta-analysis of RCTs up to July 11, 2022, showed that higher risk of any intracerebral hemorrhage in the BT group despite a higher successful recanalization rate [32].IVT, d-MT and BT, which therapy is more prone to complications leading to END, can't be generalized but should be specific according to different stroke subgroups.

This study has several limitations. First, it is a retrospective analysis and the occurrence of END of a small number of patients was mainly determined according to the course record, which has a certain bias. Second, the causes of END after BT were not categorized and the level of SBP at admission was not further stratified analyzed because of the small sample size of the present study. In the future, we will further expand the sample size for subgroup analysis and etiological analysis. Large multicenter prospective RCTs are necessary to distinguish the best beneficiaries of BT.

## CONCLUSION

The incidence of END after BT in AIS patients was 33.67%. An increase of SBP, higher glucose levels at admission and LAA were independent risk factors of END that predict a poor prognosis. Future randomized clinical trials are warranted to validate the present results.

## Figures and Tables

**Fig. (1):**
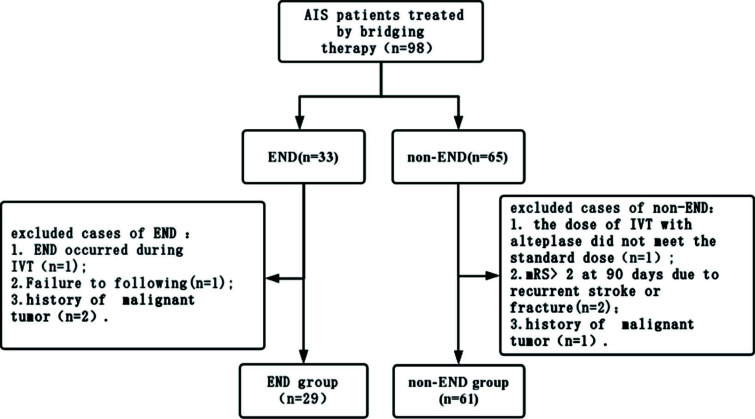
The study population flowchart. **Abbreviations:** AIS, acute ischemic stroke; END, early neurological deterioration; IVT, intravenous thrombolysis; mRS, Modified Rankin scale.

**Table 1:** Demographic and clinical data of the non-END group and the END group.

**Variable**	**Non-END Group (n=61)**	**END Group (n=29)**	***p Value***
Gender, male, n (%)	38 (62.30)	16 (55.17)	0.519
Age (year)	61.5±12.7	67.3±12.2	0.043*
Hypertension	26 (42.62)	18 (62.07)	0.085
Diabetes mellitus	6 (9.84)	8 (27.59)	0.058
Coronary artery disease	3 (4.92)	4 (13.79)	0.206
Atrial fibrillation	12 (19.67)	12 (41.38)	0.030*
Prior TIA or stroke	11 (18.03)	5 (17.24)	0.927
Current smoking	10 (16.39)	2 (6.90)	0.324
Blood glucose (mmol/l)	6.1 (5.1-7.2)	7.0 (5.6-9.4)	0.012*
White blood cells (10^9^/L)	10.20 (7.68-13.35)	9.16 (7.50-12.20)	0.424
Platelets (10^9^/L)	244.9±74.1	225.3±73.3	0.245
Fibrinogen (g/L)	2.92 (2.39-3.49)	3.16 (2.35-3.71)	0.334
INR	0.97 (0.94-1.02)	0.99 (0.97-1.03)	0.140
PT (s)	13.01±0.77	13.32±0.87	0.213
TT (s)	17.25±1.11	17.64±1.91	0.390
TG (mmol/L)	1.09 (0.87-1.51)	1.23 (0.91-1.61)	0.534
TC (mmol/L)	4.33 (3.48-4.93)	4.35 (3.28-5.23)	0.727
HDL (mmol/L)	2.70 (2.10-3.16)	2.79 (2.21-3.57)	0.400
LDL (mmol/L)	1.10±0.25	1.12±0.24	0.848
HCY (μmol/L)	10.1 (8.0-14.1)	9.8 (7.8-12.9)	0.623
SBP at admission	150.2±21.2	162.1±25.0	0.021*
DBP at admission	83.5±12.2	89.2±16.4	0.071
NIHSS score at admission	13.7±5.6	15.5±5.9	0.171
**TOAST Classification**			
LAA, n (%)	50 (81.97)	17 (58.62)	0.018*
Cardioembolism	10 (16.39)	12 (41.38)	
Others, n (%)	1 (1.64)	0	
**Occlusion Vascular Territories**			
ACS, n (%)	50 (81.97)	22 (75.86)	0.499
PCS, n (%)	11 (18.03)	7 (24.14)	
OTT (min)	139 (108-191)	140 (120-195)	0.610
OTP (min)	249.3±88.8	269.7±97.8	0.326
OTR (min)	292.0±90.7	327.6±111.6	0.120
PTR (min)	40 (30-60)	55 (31-70)	0.185
Recanalization (mTIC 2b-3), n (%)	58 (95.08)	24 (82.76)	0.055

**Note:** Values were expressed as mean±SD, median (interquartile range), or number (%); **Abbreviations:** END, early neurological deterioration; SBP, systolic blood pressure; DBP, diastolic blood pressure; TIA, transient ischemic attacks; INR, international normalized ratio; PT, prothrombin time; TT, thrombin time; TG, triglycerides; TC, total cholesterol; HDL, high-density lipoprotein; LDL, low-density lipoprotein; HCY, homocysteine; NIHSS, National Institutes of Health Stroke Scale; LAA, large artery atherosclerosis; ACS, anterior circulation stroke; PCS, posterior circulation stroke; OTT, symptom onset to thrombolysis time; OTP, symptom onset to groin puncture time; OTR, symptom onset to first recanalization time ; PTR, puncture to first recanalization time. *p < 0.05.

**Table 2:** Multivariate analysis of risk factors of END after bridging therapy.

**Variable^#^**	***P Value***	**OR**	**95%CI**
SBP at admission	0.043	1.026	1.001-1.051
Blood glucose at admission	0.007	1.389	1.092-1.176
LAA	0.001	8.009	2.357-27.223

**^#^Note:** Values were adjusted for age, LAA, history of atrial fibrillation, SBP, blood glucose at admission; **Abbreviations:** OR, odds ratio; CI, confidence interval; SBP, systolic blood pressure; LAA, large artery atherosclerosis.

**Table 3:** Clinical outcomes at 90 days of non-END group and END group.

**Indicators**	**Non-END Group (n=61)**	**END Group (n=29)**	***P Value***
mRS≤2, n (%)	40 (65.57)	2 (6.90)	0.001
mRS>2, n (%)	21 (34.43)	27 (93.10)	
Death, n (%)	3 (4.92)	13 (44.83)	0.001

**Note:** Values were expressed in number (%); **Abbreviations:** END, early neurological deterioration; mRS, modified Rankin Scale.

## Data Availability

All data generated or analyzed during this study are included in this article. Further inquiries can be directed to the corresponding author.

## LIST OF ABBREVIATIONS

ACS: Anterior Circulation Stroke
AIS: Acute Ischemic Stroke
BT: Bridging Therapy
CI: Confidence Intervals
d-MT: Direct Mechanical Thrombectomy
DBP: Diastolic Blood Pressure
END: Early Neurological Deterioration
HCY: Homocysteine
HDL: High-density Lipoprotein
INR: International Normalized Ratio
IVT: Intravenous Thrombolysis
LAA: Large Artery Atherosclerosis
LDL: Low-density Lipoprotein
LVO: Large Vessel Occlusion
MMP-9: Matrix Metalloproteinase-9
mRS: Modified Rankin Scale
MT: Mechanical Thrombectomy
ND: Neurological Deterioration
NIHSS: National Institutes of Health Stroke Scale
OR: Odds Ratios
OTP: Symptom Onset to Groin Puncture Time
OTR: Symptom Onset to First Recanalization Time
OTT: Symptom Onset to Thrombolysis Time
PCS: Posterior Circulation Stroke
PT: Prothrombin Time
PTR: Puncture to First Recanalization Time
RCTs: Randomized Controlled Trials
SBP: Systolic Blood Pressure
SD: Standard Deviation
sICH: Symptomatic Intracranial Hemorrhage
TC: Total Cholesterol
TG: Triglycerides
TIA: Transient Ischemic Attacks
TT: Thrombin Time

## STANDARDS OF REPORTING

STROBE guidelines were followed.
